# Hydrogen Peroxide Signaling in Plant Development and Abiotic Responses: Crosstalk with Nitric Oxide and Calcium

**DOI:** 10.3389/fpls.2016.00230

**Published:** 2016-03-04

**Authors:** Lijuan Niu, Weibiao Liao

**Affiliations:** Department of Ornamental Horticulture, College of Horticulture, Gansu Agricultural UniversityLanzhou, China

**Keywords:** hydrogen peroxide (H_2_O_2_), nitric oxide (NO), calcium (Ca^2+^), signal molecule, crosstalk

## Abstract

Hydrogen peroxide (H_2_O_2_), as a reactive oxygen species, is widely generated in many biological systems. It has been considered as an important signaling molecule that mediates various physiological and biochemical processes in plants. Normal metabolism in plant cells results in H_2_O_2_ generation, from a variety of sources. Also, it is now clear that nitric oxide (NO) and calcium (Ca^2+^) function as signaling molecules in plants. Both H_2_O_2_ and NO are involved in plant development and abiotic responses. A wide range of evidences suggest that NO could be generated under similar stress conditions and with similar kinetics as H_2_O_2_. The interplay between H_2_O_2_ and NO has important functional implications to modulate transduction processes in plants. Moreover, close interaction also exists between H_2_O_2_ and Ca^2+^ in response to development and abiotic stresses in plants. Cellular responses to H_2_O_2_ and Ca^2+^ signaling systems are complex. There is quite a bit of interaction between H_2_O_2_ and Ca^2+^ signaling in responses to several stimuli. This review aims to introduce these evidences in our understanding of the crosstalk among H_2_O_2_, NO, and Ca^2+^ signaling which regulates plant growth and development, and other cellular and physiological responses to abiotic stresses.

## Introduction

Hydrogen peroxide (H_2_O_2_), a form of reactive oxygen species, is regarded as a common cellular metabolite. H_2_O_2_ is continually synthesized through various sources including enzyme and non-enzyme pathways in plants. To date, it has become accepted that H_2_O_2_ plays important roles in plant developmental and physiological processes including seed germination (Barba-Espín et al., 2011), programmed cell death (PCD; Cheng et al., 2015; Vavilala et al., 2015), senescence (Liao et al., 2012b), flowering (Liu et al., 2013), root system development (Liao et al., 2009; Ma et al., 2014; Hernández-Barrera et al., 2015), stomatal aperture regulation (Ge et al., 2015) and many others. It is now clear that H_2_O_2_ functions as a signaling molecule which may respond to various stimuli in plant cells. These results suggest that H_2_O_2_ may be involved in cellular signaling transduction pathways and gene expression modulations in plants.

Nitric oxide (NO), as a small signaling molecule, appears to be involved in plant developmental and physiological processes such as seed germination (Wang et al., 2015), ripening and senescence (Shi Y. et al., 2015) as well as stomatal closure (Shi K. et al., 2015) and pollen tube growth (Wang et al., 2009). Meanwhile, NO signaling may have a vital role in the disease resistance (Kovacs et al., 2015) and response to abiotic stresses such as cold (Fan et al., 2015), salt (Liu W. et al., 2015) and drought (Shan et al., 2015). Calcium ion (Ca^2+^) signaling is also a core regulator of plant physiological process and stress adaption such as cell polarity regulation (Zhou et al., 2014), leaf de-etiolation (Huang et al., 2012), stomatal closure (Zou et al., 2015). Additionally, Ca^2+^ signaling is also involved in various responses to abiotic stimuli, including light (Hu et al., 2015) and heavy metal (Li et al., 2016).

A large amount of research show that H_2_O_2_, NO and Ca^2+^ as signaling are involved in plant growth and development as well as response to abiotic stresses. In this review, we focus on H_2_O_2_ signaling activities and its cross-talk with Ca^2+^ and NO in plants.

## H_2_O_2_ homeostasis

### H_2_O_2_ generation

**Figure 1:**
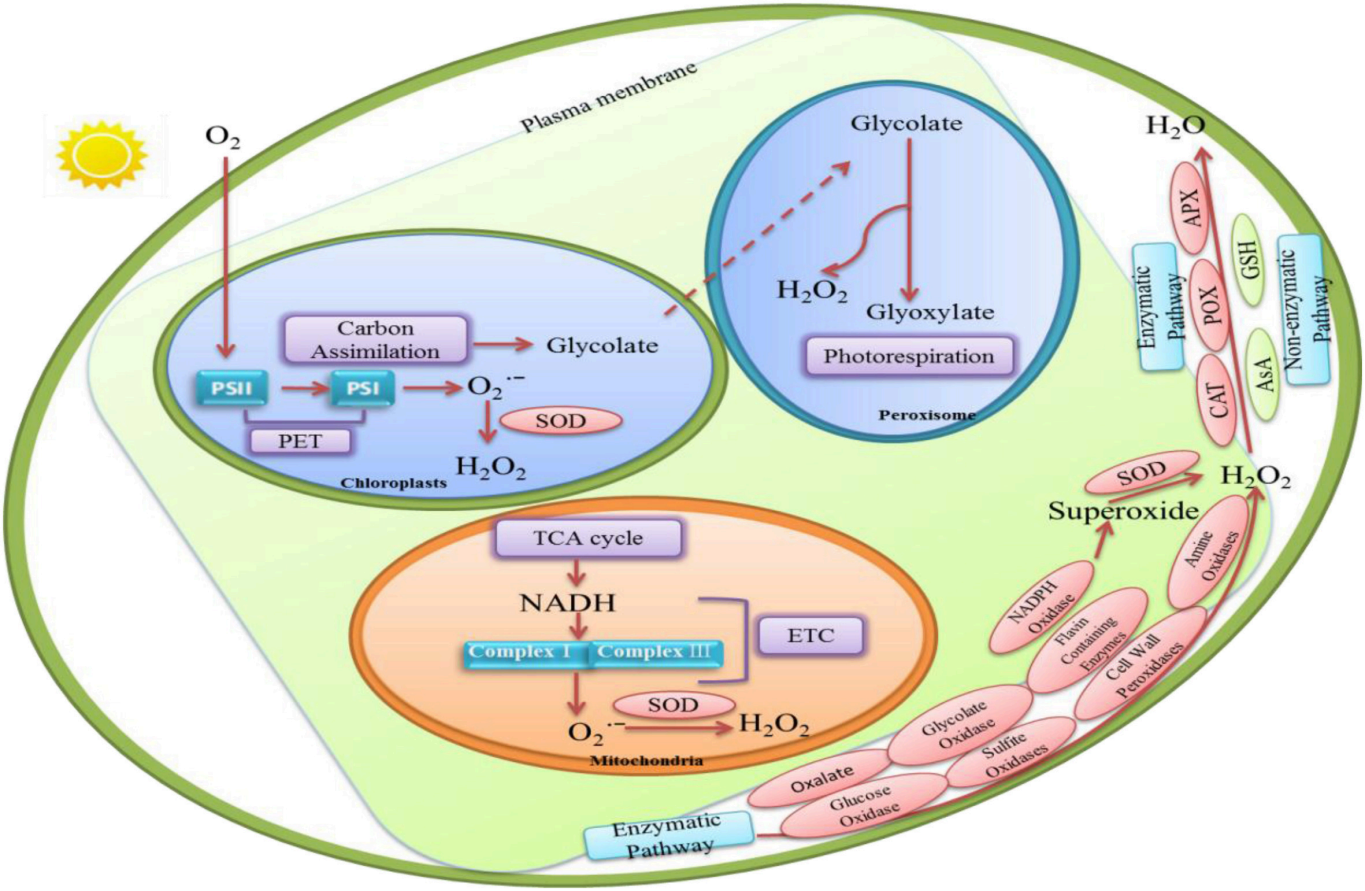
**The various routes of hydrogen perioxide (H_2_O_2_) production and H_2_O_2_ removal in plant cells**. Enzymatic production of H_2_O_2_ in plants requires several enzymes including cell wall peroxidases (Francoz et al., 2015), oxalate (Hu et al., 2003), amine oxidases and flavin-containing enzymes (Cona et al., 2006), glucose oxidases, glycolate oxidases (Chang and Tang, 2014), and sulfite oxidases (Brychkova et al., 2012). In these enzymes, some of them may convert O${}_{2}^{-}$ to H_2_O_2_ and O_2_. And others may oxidize their each substrates to generate H_2_O_2_in biocatalysis processes. Several non-enzymatic reactions are also known to produce H_2_O_2_. In peroxisome, H_2_O_2_ synthesis is associated with glycolate oxidation during photosynthetic carbon oxidation cycle (Foyer and Noctor, 2003). In chloroplasts, H_2_O_2_ production can be produced by the reduction of O${}_{2}^{-}$ by photosynthetic electron transport (PET) chain. H_2_O_2_ in chloroplast also may be detected at the manganese-containing, oxygen evolving complex which is the donor site of photosystem II. Moreover, H_2_O_2_ could be generated in mitochondria through aerobic respiration because O${}_{2}^{-}$ is produced from complexes I and III in the electron transport chain. H_2_O_2_-scavenging enzymes include catalase (CAT; Willekens et al., 1997), peroxidase (POX; Fan and Huang, 2012), ascorbate peroxidase (APX) and glutathione reductase (GR; Jahan and Anis, 2014). In non-enzymatic pathway, Ascorbate (AsA) and glutathione (GSH) are responsible for decreasing H_2_O_2_ level (Kapoor et al., 2015).

H_2_O_2_ is a byproduct of aerobic metabolism in plants (Mittler, 2002). Figure 1 shows that H_2_O_2_ in plants can be synthesized either enzymatically or non-enzymatically. There are numerous routes of H_2_O_2_ production in plant cells, such as photorespiration, electron transport chains (ETC), and redox reaction.

There is evidence for H_2_O_2_ production in plants through several enzymes includingcell wall peroxidases (Francoz et al., 2015), oxalate (Hu et al., 2003), amine oxidases and flavin-containing enzymes (Cona et al., 2006; Figure 1). Moreover, nicotinamide adenine dinucleotide phosphate (NADPH) oxidases may also increase H_2_O_2_ level through generating superoxide which could be converted to H_2_O_2_ by superoxide dismutases (SOD; Grivennikova and Vinogradov, 2013; Brewer et al., 2015). Remans et al. (2010) observed that ROS accumulation, especially H_2_O_2_ formation, is mostly related with the stimulation of NADPH oxidase in plants under heavy metal stresses. Moreover, H_2_O_2_ produced by NADPH oxidases may significantly increase proline accumulation in *Arabidopsis thaliana* under salt or mannitol stress (Ben Rejeb et al., 2015). Additionally, some other oxidases such as glucose oxidases, glycolate oxidases (Chang and Tang, 2014), and sulfite oxidases (Brychkova et al., 2012) may oxidize their own substrates to produce H_2_O_2_ (Figure 1).

Several non-enzymatic reactions are also known to produce H_2_O_2_. For example, many reactions involved in photosynthesis and respiration are responsible for H_2_O_2_ production. It is generated continually via electron transport reactions both in mitochondria and chloroplasts (Figure 1).

#### Peroxisomes

Peroxisome is considered to be the site of photorespiration in plant cell, which needs light-dependent uptake of O_2_ and releases CO_2_ accompanying with the generation of H_2_O_2_. It is suggested that H_2_O_2_ synthesis is associated with the oxidation of glycolate during the photosynthetic carbon oxidation cycle (Foyer and Noctor, 2003; Figure 1).

#### Chloroplasts

Chloroplast is the source of photosynthesis in plants. Chloroplasts are the crucial sites for H_2_O_2_ production during photosynthesis. H_2_O_2_ generation is associated with oxygen reduction in chloroplast (Figure 1). Mehler (1951) discovered that reduction of O_2_ lead to the formation of H_2_O_2_ in the presence of light in chloroplast. Moreover, H_2_O_2_ production can also be produced by the reduction of O${}_{2}^{\overset{{}^{\circ}}{-}}$ by photosynthetic electron transport (PET) chain components such as Fe–S centers, reduced thioredoxin (TRX), ferredoxin and reduced plastoquinone in the chloroplast (Dat et al., 2000). In addition, non-enzymatic production of H_2_O_2_ in chloroplast may be detected at the manganese-containing, oxygen evolving complex which is the donor site of photosystem II (Figure 1). But this process, in most cases, may probably be ignored under physiological conditions.

#### Mitochondria

One important source of endogenously produced H_2_O_2_ in plant cell is mitochondria (Dickinson and Chang, 2011). H_2_O_2_ is generated in mitochondria during aerobic respiration when O${}_{2}^{-}$ is produced from complexes I and III in the electron transport chain, which is then rapidly converted to H_2_O_2_ by the enzyme superoxide dismutase (Figure 1).

### H_2_O_2_ removal

The antioxidant systems that regulate H_2_O_2_ levels consist of both non-enzymatic and enzymatic H_2_O_2_ scavengers (Figure 1). H_2_O_2_-scavenging enzymes include catalase (CAT; Willekens et al., 1997), peroxidase (POX; Fan and Huang, 2012), ascorbate peroxidase (APX) and glutathione reductase (GR; Jahan and Anis, 2014). Some studies revealed that APX was found in the cytosol (Begara-Morales et al., 2013), chloroplasts (Asada, 2006), and mitochondria (Navrot et al., 2007). Meanwhile, CAT can decompose H_2_O_2_ in peroxisome (Nyathi and Baker, 2006). It is quite clear that these enzymes exist in different organelles and they might decrease H_2_O_2_ content efficiently and maintain the stability of membranes.

Ascorbate (AsA) and glutathione (GSH), as non-enzymatic compounds, are constantly participated in regulating ROS level (Kapoor et al., 2015). AsA, a key antioxidant for elimination of H_2_O_2_, can react with H_2_O_2_ directly. GSH is a crucial antioxidant which may be associated with regenerating AsA, and rapidly oxidizes excess H_2_O_2_. Therefore, GSH is also involved in regulating H_2_O_2_ level and redox balance in plant cells (Krifka et al., 2012). In fact, H_2_O_2_ homeostasis seems to result in some biological effects on plant cells which may be as a signaling sign in signaling transduction pathway.

### Responses to H_2_O_2_

#### Growth and development

Table 1 shows that H_2_O_2_ mediates various developmental and physiological processes in plants. These findings indicate that H_2_O_2_ may affect different parts of plants by increasing endogenous H_2_O_2_ level or by regulating relative gene expression. Also, the change of H_2_O_2_ level may impact metabolic and antioxidant enzyme activity in favor of plant growth and development (Barba-Espín et al., 2011; Liu et al., 2013). However, the mechanisms that allow different H_2_O_2_ function in plants still require examination.

**Table 1:** **The developmental and physiological effects of H_2_O_2_ in plants**.

**Developmental and physiological effect**	**Species**	**Tissue**	**H_2_O_2_ production**	**H_2_O_2_-mediated effect**	**References**
Seed germination	*Pisum sativum* L. cv. Alaska	Seed	+	Caused carbonylation of proteins and metabolic enzyme Up-regulated *PsMAPK2* *PsMAPK3* expression	Barba-Espín et al., 2011
PCD	*Triticum aestivum* L.	Seedling	+	Increased antioxidant enzyme activities and gene expression	Cheng et al., 2015 Vavilala et al., 2015
	*Chlamydomo-nas reinhardtii*		+	Induced cell death Increased intracellular H_2_O_2_ content Increased antioxidant enzyme activities and analyses of transcripts	
Senescence	*Lilium*	Leaf	+	Increased vase life and flower diameter Reduced the degradation of RWC, total chlorophyll content and water-soluble carbohydrate	Liao et al., 2012b
Flowering	*Monilinia fructicola*	Petal	+	Increased H_2_O_2_ concentration Enhanced protein carbonylation (carbonyl content)and lipid peroxidation (MDA content)	Liu et al., 2013
Root system development	*Tagetes erecta* L.	Root	+	Increased root length Increased root number explant^−1^	Liao et al., 2009
	*Arabidopsis thaliana*			Accelerated lateral root formation Increased endogenous H_2_O_2_ production Up-regulated relative expression levels of *HY1*	Ma et al., 2014
				Increased sensitivity of the root elongation zone	Hernández-Barrera et al., 2015
Stomatal closure	*Arabidopsis thaliana*	Leaf	+	Induced stomatal closure	Ge et al., 2015

#### Stress condition

Recent studies have demonstrated that H_2_O_2_ is a key signaling molecule in the signaling pathway, which associated with abiotic stress response. A number of discussions showed that H_2_O_2_ could respond to abiotic stresses such as drought (Hameed and Iqbal, 2014; Ashraf et al., 2015), salinity (Sathiyaraj et al., 2014; Mohamed et al., 2015), cold (Orabi et al., 2015), high temperatures (Wang Y. et al., 2014; Wu et al., 2015), UV radiation (He et al., 2005), ozone (Oksanen et al., 2004), and heavy metal (Wen et al., 2013; Table 2). It is clear from these studies that H_2_O_2_ could enhance abiotic stress resistance through protecting organelle structure under abiotic stress conditions. For instance, H_2_O_2_ may protect chloroplast ultrastructure to preserve photosynthesis under abiotic stress. Similarly, to improve plant abiotic stress tolerance, H_2_O_2_ may modulate the expression of resistance genes and antioxidant enzyme activities during abiotic stress response.

**Table 2:** **Report on H_2_O_2_-mediated effect during stresses in plants**.

**Stress**	**Plant species**	**Tissue**	**H_2_O_2_-mediated effect**	**References**
Drought	*Triticum aestivum* L.	Leaf	Increased SOD, POD, CAT activities Raised total phenolic and reducing sugars content	Hameed and Iqbal, 2014
	*Zea mays* L.	Leaf	Reduced degradation of chlorophyll increased endogenous H_2_O_2_, MDA contents Increased antioxidant enzymes activities Increased ascorbic acid content and ion contents	Ashraf et al., 2015
Salt	*Panax ginseng*	Leaf	Increased chlorophyll and carotenoid content Increased Relative water content Increased growth height and dry-weight Increased antioxidant activity Up-regulated relative gene expression of defense related genes	Sathiyaraj et al., 2014
	*Lycopersicon esculentum* L.		Decreased electrolyte leakage Increased endogenous H_2_O_2_ and MDA content Increased antioxidant enzymes activities Affect protein pattern and peroxidase enzymes	Mohamed et al., 2015
Cold	*Lycopersicon esculentum* L.	Seedling	Increased antioxidant enzymes activities Increased MDA content Decreased electrolyte leakage Increased total soluble solids	Orabi et al., 2015
Heat	*Festuca arundinacea Lolium perenne*	Leaf	Decreased the GSH/GSSG ratio Increased POD, CAT, APC, GR, and GPX activities	Wang Y. et al., 2014
	*Arabidopsis thaliana*	Seedling	Increased thermotolerance Enhanced antioxidant enzyme activities Increased endogenous NO content Increased HSFs activity and HSP21 accumulation	Wu et al., 2015
UV-B	*Vicia faba* L.	Leaf	Increased endogenous H_2_O_2_ production Induced Stomatal closure	He et al., 2005
Ozone	*Betula papyrifera*	Leaf	Induced proliferation of peroxisomes Increased Level of gene expression for catalase (*Cat*)	Oksanen et al., 2004
Heavy metal	*Zea mays var*. rugosa Bonaf	Seedling	Decreased the activities of proline dehydrogenase Increased the activities of Arginase and OAT, P5CS and GDH Up-regulated the expression levels of *P5CS, GDH, Arginase, OAT* and *ProDH* genes	Wen et al., 2013

### H_2_O_2_ as a signaling molecule in plant

Among ROS, H_2_O_2_ has comparatively long life span and small size, which permit it to traverse through cellular membranes to different cellular compartments. García-Mata and Lamattina (2013) found that H_2_O_2_ may move between cells through aquaporin channels for signaling transduction. Increasing evidences point out that H_2_O_2_ signaling may regulate various plant physiological processes. For example, H_2_O_2_ as signaling molecule may participate in nitrosative stress-triggered cell death in kimchi cabbage (*Brassica rapa* var. *glabra* Regel) seedlings (Kim et al., 2015). Also, Li et al. (2015) suggested that H_2_O_2_ is involved in signaling crosstalk between NO and hydrogen sulfide (H_2_S) to induce thermotolerance in maize seedlings. Moreover, the interaction among H_2_O_2_, NO and Ca^2+^ could relieve copper stress in *Ulva compressa* (González et al., 2012). H_2_O_2_ signaling was also demonstrated to play a salient role in brassinosteroid-regulated stomatal movement (Shi C. et al., 2015). As stated above, H_2_O_2_ as an important signaling molecule may play a significant role at every stage of plant life and under various abiotic stress conditions. H_2_O_2_ signaling appears to crosstalk with many different signaling molecules such as hormones (Shi C. et al., 2015), protein kinase (González et al., 2012) and many other small signaling molecules (Li et al., 2015). H_2_O_2_ and these signaling molecules may influence each other through various positive and negative feedback loops. Thus, they co-regulate cell division and differentiation, antioxidant system as well as gene expression involved in plant development and defense.

## Crosstalk between H_2_O_2_ and No

**Figure 2:**
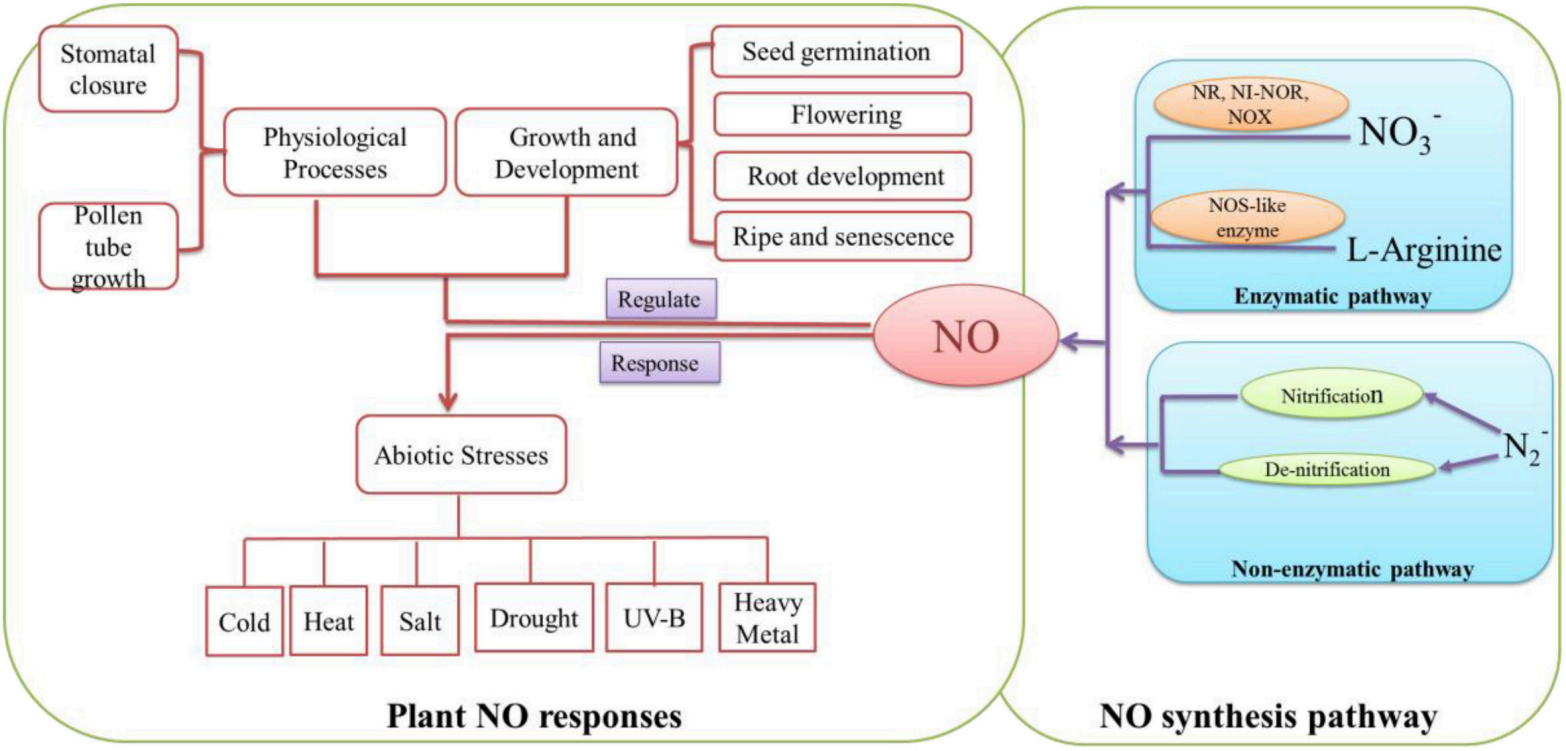
**Summary of the main NO systhetic pathways and NO functions in plant growth, development and defense processes**. NO may be synthesized by enzymatically and non-enzymatically pathways. In enzymatic pathway, nitrate reductase (NR; Rockel et al., 2002), Nitrite-NO reductase (Ni-NOR; Stöhr et al., 2001) and xanthine oxidase (XOR; Corpas et al., 2004) could convert NO${}_{3}^{-}$ and NO${}_{2}^{-}$ to NO. Meanwhile, because of NOS-like enzyme (Guo et al., 2003), L-Arginine may be catalyzed to NO. In non-enzymatic pathway, N${}_{2}^{-}$ could be transformed to NO through nitrification and denitrification (Skiba et al., 1993). NO plays an important signaling molecule in plant. It could regulate developmental and physiological processes such as seed germination (Wang et al., 2015), root development (Liao et al., 2011) and stomatal closure (Shi C. et al., 2015). Also, it may be involved in response to abiotic stresses such as cold (Fan et al., 2015), salt (Liu W. et al., 2015) and drought (Shan et al., 2015).

NO is a diatomic free radical gas. Previous studies suggested that NO could take part in a wide range of physiological processes such as vasorelaxation, nervous system, defense against pathogens in animals (Mayer and Hemmens, 1998). In mammals, NO is synthesized via three different isoforms of NO synthase (NOS) including inducible NOS (iNOS; Nathan and Hibbs, 1991), endothelial NOS (eNOS) and neuronal NOS (nNOS; Förstermann et al., 1994). In plants, NO could be synthesized through enzymatic and non-enzymatic pathways (Figure 2). The enzymatic pathway includes nitrate reductase (NR; Rockel et al., 2002), nitric oxide-like (NOS-like) synthase (Guo et al., 2003), Nitrite-NO reductase (Ni-NOR; Stöhr et al., 2001) and xanthine oxidase (XOR; Corpas et al., 2004) pathways. The non-enzymatic generation of NO includes nitrification or de-nitrification processes (Skiba et al., 1993, Figure 2).

A plethora of evidences suggest that NO, as a versatile signaling molecule, is involved in regulating every aspect of plant growth and developmental processes such as seed germination (Fan et al., 2013; Wang et al., 2015), flowering (Liu W. W. et al., 2015), root growth and development (Liao et al., 2011; Wu et al., 2014; Xiang et al., 2015), ripening and senescence (Liao et al., 2013; Shi Y. et al., 2015). Meanwhile, as a physiological regulator, NO signaling is involved in mediating stomatal closure (Noelia et al., 2015; Shi K. et al., 2015; Chen et al., 2016), pollen tube growth (Wang et al., 2009). Also, NO plays an essential role in plant disease resistance (Rasul et al., 2012; Kovacs et al., 2015) and responses to various abiotic stresses such as cold (Fan et al., 2015), heat (Yu et al., 2015), salt (Liu W. et al., 2015), drought (Shan et al., 2015), UV-B (Esringu et al., 2015) and heavy metal (Alemayehu et al., 2015; Chen et al., 2015; Kaur et al., 2015). These studies have paved the way to understand the signaling roles of NO which may affect cell metabolism, cellular redox balance and gene expression in plants. The relative target receptor may receive signaling activated by various stimuli. As a result, NO may activate regulatory mechanism to promote developmental and physiological processes and regulate abiotic stress response in plants.

### Interaction in growth and development

To date, the interaction between H_2_O_2_ and NO has been demonstrated clearly in plants. The signaling crosstalk between H_2_O_2_ and NO has been considered to be an essential factor to influence plant developmental and physiological processes such as leaf cell death (Lin et al., 2012), delay senescence (Iakimova and Woltering, 2015), root growth and development (Liao et al., 2010, 2011), stomatal closure (Huang et al., 2015; Shi K. et al., 2015), and pollen tube growth (Serrano et al., 2012). Table 3 shows the interaction of H_2_O_2_ and NO at different levels in a great number of developmental and physiological processes in plants. On the one side, H_2_O_2_ may act as a cofactor to promote endogenous NO synthesis. For example, Lin et al. (2012) implied that H_2_O_2_ may stimulate NO production through increasing NR activity in leaves of *noe1* plants under high light. Shi C. et al. (2015) reported that Gα-activated H_2_O_2_ production may induce NO synthesis. The research found that NO could modulate stomatal closure in H_2_O_2_ mutants *AtrbohF* and *AtrbohD AtrbohF* and in the wild type treated with H_2_O_2_ scavenger and inhibitor. However, H_2_O_2_ did not close or reduce the stomatal closure in mutants *Nia1-2* and *Nia2-5 Nia1-2*, and in the wild type treated c-PTIO or tungstate (Shi C. et al., 2015). These results clearly show that H_2_O_2_ might induce NO synthesis in stomatal closure. On the other side, NO may induce H_2_O_2_ generation in plants. Liao et al. (2011) reported cPTIO or L-NAME could inhibit the endogenous H_2_O_2_ generation implying that NO was required for the production of H_2_O_2_ during adventitious rooting. Meanwhile, NO could mediate antioxidant enzyme activities to influence the H_2_O_2_ level (Zhang et al., 2007). Thus, the interaction of H_2_O_2_ and NO may trigger a serious of physiological and biological response in plant cells.

**Table 3:** **The developmental and physiological effects of crosstalk between H_2_O_2_ and NO in plants**.

**Developmental and physiological effect**	**Species**	**Tissue**	**Crosstalk between H_2_O_2_ and NO mediated effects**	**References**
Cell death	*Oryza sativa*	Leaf	H_2_O_2_ induced NR-dependent NO generation NO Is required for H_2_O_2_-induced leaf cell death increased NR enzyme	Lin et al., 2012
Senescence	*Lactuca sativa* L.	Leaf	NO decreased endogenous H_2_O_2_ content Delay senescence	Iakimova and Woltering, 2015
Root growth	*Dendranthema morifolium*	Root	Increase the activities of PPO, IAAO and the content of WSC and total nitrogen Decrease the total polyphenol content NO and H_2_O_2_ may act synergistically to mediate adventitious root generation and development	Liao et al., 2010 Liao et al., 2011
	*Tagetes erecta* L.		NO may be involved as an upstream signaling molecule for H_2_O_2_ production	
Pollen tube growth	*Olea europaea* L.	Flower	Decreased cell death Increased nitrated proteins	Serrano et al., 2012
Stomatal movement	*Arabidopsis*	Leaf	H_2_O_2_ production was required for NO synthesis Regulated stomatal closure	Shi C. et al., 2015 Huang et al., 2015
	*Vicia faba*		Regulated stomatal closure H_2_O_2_ induced NO production	

### Interaction during abiotic stress

Recently, the roles of H_2_O_2_and NO signaling and their crosstalk in mediating plant response to abiotic stresses have been largely established (Table 4).

**Table 4:** **Reports on interaction between H_2_O_2_ and NO involved in abiotic stresses in plants**.

**Stress**	**Plant species**	**Tissue**	**Crosstalk between H_2_O_2_ and NO mediated effects**	**Reference**
Salt	*Citrus aurantium* L.	Leaf	Alleviated salinity-induced protein carbonylation Shifted the accumulation levels of leaf S-nitrosylated proteins acclimation to salinity Identified a number of proteins which were modulated by both H_2_O_2_ and NO treatments	Tanou et al., 2009 Tanou et al., 2010
	*Populus euphratica*	shoot	Increased K/Na ratio	Zhang et al., 2007
	*Medicago falcata*	Seedling	Stimulated expression of PM H^+^-ATPase Induced *MfMIPSI* transcript Increased the level of myo-inositol	Tan et al., 2013
Drought	*Tagetes erecta* L.	Root	Alleviated the destruction of mesophyll cell ultrastructure Increased leaf chlorophyll content Mediated chlorophyll fluorescence parameters Enhanced carbohydrate accumulation Decreased starch content H_2_O_2_ generation may be affected by NO	Liao et al., 2012a
	*Tagetes erecta* L.	Leaf	Increased RWC Decrease ion leakage Increased antioxidant enzyme, PEPCase, HK activities and MDA content NO acted downstream of H_2_O_2_	Lu et al., 2009
UV-B	*Arabidopsis*	Leaf	NO production depends on H_2_O_2_ Mediated stomatal closure	He et al., 2013
			The UV-B Photoreceptor UVR8 was mediated by H_2_O_2_ and NO	Tossi et al., 2014
Heat	*Zea may* L.	seedling	Improved survival percentage of maize seedlings H_2_O_2_ increased endogenous NO content H_2_O_2_ may be involved in downstream signal of NO	Li et al., 2015
	*Arabidopsis*		NO is involved in H_2_O_2_ signaling as a downstream factor. Increased HS factor activity and HS protein accumulation.	Wang L. et al., 2014
	*Triticum aestivum* L.		Increased seedling resistance Increased H_2_O_2_ and NO content Increased survival percentage of seedlings	Karpets et al., 2015
Cold	*Medicago sativa* subsp. falcata	Leaf	Mediated cold-induced *MfSAMS1* expression	Guo et al., 2014
	*Medicago falcate*	Seedling	Up-regulated *MfMIPSI* expression	Tan et al., 2013
	*Medicago sativa*		Increased myo-inositol content	
Heavy metal	*Ulva compressa*	Cell	Increased PDH,IDH,OGDH activity and increased relative transcript levels	González et al., 2012
	*Triticum aestivum*	Root	Decreased lipid peroxidation Increased NOS activity Increased antioxidative enzyme activities	Duan et al., 2015

#### Drought

Drought stress is a major environmental factor that affects plant growth and development. As reported by Liao et al. (2012a), both H_2_O_2_ and NO could protect mesophyll cells ultrastructure and improve the photosynthetic level of leaves under drought stress during adventitious rooting in marigold explants. Similarly, the interplay between H_2_O_2_ and NO signaling may increase the activity of myo-inositol phosphate synthase to alleviate drought stress (Tan et al., 2013). Additionally, Lu et al. (2009) suggested that endogenous NO and H_2_O_2_ may be involved in ABA-induced drought tolerance of bermudagrass by increasing antioxidant enzyme activities. NO may be considered to be upstream or downstream signaling molecule of H_2_O_2_ (Lu et al., 2009; Liao et al., 2012a). Thus, the interaction between H_2_O_2_ and NO may alleviate drought stress through up-regulating antioxidant defense system to protect cell membrane and maintain ion homeostasis in plants.

#### Salt

The interaction between H_2_O_2_ and NO plays an important role in plant tolerance to salt stress (Zhang et al., 2007; Tan et al., 2013). Tanou et al. (2009) suggested that H_2_O_2_ and NO pre-treatments could alleviate salinity-induced protein carbonylation in citrus. The authors suggested an interaction between H_2_O_2_ and NO during salt stress response. Furthermore, H_2_O_2_- and NO-responsive proteins have been identified which may further reveal a protein interaction network between H_2_O_2_ and NO signaling under salt stress (Tanou et al., 2010).

#### UV-B

UV-B, a key environmental signal, initiates diverse responses in plants (Jansen and Bornman, 2012). UV-B radiation can also influence plant growth, development, and productivity. It has been shown that the crosstalk between H_2_O_2_ and NO could be involved in the response to UV-B stress. There was an interrelationship among Gα protein, H_2_O_2_, and NO during UV-B-induced stomatal closure in *Arabidopsis* leaves (He et al., 2013). This study found that there was a significant increase in H_2_O_2_ or NO levels which associated with stomatal closure in the wild type by UV-B stress. However, these effects were abolished by double mutants of *AtrbohD* and *AtrbohF* or *Nia*1 mutants. These results strongly suggested that the crosstalk between H_2_O_2_ and NO signaling might play an essential role during UV-B-induced stomatal closure in guard cells. Recently, Tossi et al. (2014) also showed a mechanism involving both H_2_O_2_ and NO generation in response to UV-B exposure. Therefore, the crosstalk between H_2_O_2_ and NO can regulate stomatal movement to reduce UV-B stress damage to plant cells.

#### Cold

Cold stress adversely influences plant growth and development. Guo et al. (2014) reported that the interaction of H_2_O_2_ and NO may affect cold-induced S-adenosylmethionine synthetase and increase cold tolerance through up-regulating polyamine oxidation in *Medicago sativa* subsp. *falcate*. Moreover, signaling interplay of H_2_O_2_ and NO was essential for cold-induced gene expression of falcata myo-inositol phosphate synthase (*MfMIPS*), which improved tolerance to cold stress (Tan et al., 2013). Thus, the interaction between H_2_O_2_ and NO may initiate different mechanisms to response to cold stresses.

#### Heat

Recently, many studies have been conducted to investigate the relationship between H_2_O_2_ and NO under heat stress. Li et al. (2015) reported that a signaling crosstalk between H_2_O_2_ and NO may be involved in inducing thermotolerance in maize seedlings. Moreover, H_2_O_2_ may be upstream signaling of NO in the heat shock pathway in *Arabidopsis* seedlings (Wang L. et al., 2014). In addition, treatment with low level of H_2_O_2_ or NO could increase seedling viability under heat resistance (Karpets et al., 2015). These studies support the existence of crosstalk between H_2_O_2_ and NO in heat responses in plants.

#### Heavy metal stress

Alberto et al. (2012) suggested that the signaling interaction between H_2_O_2_ and NO was involved in alleviating copper stress of *Ulva compressa* through mediating antioxidant enzyme activities and activating relative gene expression. Besides, the interplay of NO and H_2_O_2_ in wheat seedlings participated in regulating root growth under zinc stress and alleviated zinc stress through increasing antioxidant system, decreasing lipid peroxidation as well as up-regulating resistance gene expression (Duan et al., 2015). Obviously, the crosstalk of H_2_O_2_ and NO has been found under heavy metal stress condition, which may trigger a variety of antioxidant responses in plants.

As stated above, the physiological effect of H_2_O_2_ and NO is similar and synergetic. In different cases, these forms of interaction are various. However, the form of H_2_O_2_ and NO crosstalk depend on plant species and environmental stresses. H_2_O_2_ and NO could modulate each other through regulating antioxidant enzymes activities and relative gene expression in plants. Meanwhile, H_2_O_2_ and NO may synergistically regulate many common target genes which were related to signaling transduction, defense reaction, plant hormone interactions, protein transport and metabolism. Therefore, it has a significant meaning to elaborate the mechanism of the interaction between H_2_O_2_ and NO in plant developmental processes and response to abiotic stresses.

## Crosstalk between H_2_O_2_ and Ca^2+^

Ca^2+^ is a widespread signaling molecule in plants. When plants receive stimuli, the change of intracellular Ca^2+^ concentration may transfer signaling to regulate a series of cellular processes in plants (Kong et al., 2015; Tang et al., 2015). There are various types of Ca^2+^ receptors and channels in plants such as Ca^2+^-ATPases (Pászty et al., 2015), Ca^2+^-binding sensor protein (Wagner et al., 2015), inositol-1,4,5-trisphosphate (IP_3_; Serrano et al., 2015) and cyclic ADP-ribose (cADPR, Gerasimenko et al., 2015). It is well known that Ca^2+^ is involved in plant growth and development such as seed germination (Kong et al., 2015), pollen tube growth (Zhou et al., 2014), leaf de-etiolation (Huang et al., 2012), root growth and development (Liao et al., 2012a; Han et al., 2015) and other physiological processes including cell polarity regulation (Zhou et al., 2014; Himschoot et al., 2015), stomatal closure (Zou et al., 2015) and immune response (Seybold et al., 2014). Furthermore, variations in cytosolic free Ca^2+^ concentration have been demonstrated to response to a wide range of environmental stresses such as heat shock (Urao et al., 1994), drought (Zou et al., 2015), light (Hu et al., 2015), salt (Tepe and Aydemir, 2015), and heavy metal (Li et al., 2016). Because of Ca^2+^ has various receptors and channels in plants, it may receive different upstream signaling molecules quickly and then respond to abiotic stress.

### Interaction in growth and development

Crosstalk between H_2_O_2_ and Ca^2+^ occurs in plant cells (Table 5). For example, exogenous H_2_O_2_ caused transiently dose-dependent increase in Ca^2+^ influx in *Arabidopsis thaliana* root epidermis (Demidchik et al., 2007). Two Ca^2+^ channels could be regulated by H_2_O_2_ level in root elongation zone. Han et al. (2015) demonstrated that H_2_O_2_ signaling could induce root elongation by mediating Ca^2+^ influx in the plasma membrane of root cells in *Arabidopsis* seedlings. Richards et al. (2014) also suggested that Annexin 1, a Ca^2+^ transport protein, may regulate H_2_O_2_-induced Ca^2+^ signature in *Arabidopsis thaliana* roots to promote root growth and development. Additionally, Ca^2+^ signaling was involved in H_2_O_2_-induced adventitious rooting in marigold because removal of Ca^2+^ could inhibit H_2_O_2_-induced adventitious root development (Liao et al., 2012a). Interestingly, Wu et al. (2010)'s findings strongly suggested that spermidine oxidase (Spd)-derived H_2_O_2_ signaling may mediate Ca^2+^ influx. Spd was probably related to downstream induction of H_2_O_2_ signaling and then H_2_O_2_ activated Ca^2+^-permeable channels during pollen tube growth (Wu et al., 2010). Cross talk between Ca^2+^–Calmodulin (CaM) and H_2_O_2_ also played a significant role in antioxidant defense in ABA signaling in maize leaves (Hu et al., 2007; Table 5). Thus, the signaling crosstalk between H_2_O_2_ and Ca^2+^ may affect every stage of plant development by modulating cell elongation and division, antioxidant enzyme activity and gene expression. H_2_O_2_ may activate Ca^2+^ receptors and target proteins to increase [Ca^2+^]_cyt_ level and Ca^2+^ may induce endogenous H_2_O_2_ generation during plant growth and development.

**Table 5:** **The developmental and physiological effects of crosstalk between H_2_O_2_ and Ca^2+^ in plants**.

**Developmental and physiological effect**	**Species**	**Tissue**	**Crosstalk between H_2_O_2_ and Ca^2+^ mediated effects**	**References**
Root growth and elongation	*Arabidopsis*	Root	H_2_O_2_ induce Ca^2+^ influx Increased root elongation Endogenous H_2_O_2_ resulted in Ca^2+^ flux Enhanced root growth	Han et al., 2015 Demidchik et al., 2007
Adventitious root development	*Arabidopsis*	Root	Extracellular H_2_O_2_ induced a sustained increase in cytosolic free Ca^2+^ Exogenous H_2_O_2_ induced expression of *AtANN1*	Richards et al., 2014 Liao et al., 2012a
	*Tagetes erecta* L.		Endogenous H_2_O_2_ increased Cytosolic free Ca^2+^ and CaM content Induced adventitious root development	
Pollen growth	*P.Dyrifolia Nakai* cv.Hosui Imamuraaki	Flower	H_2_O_2_ activates Ca^2+^ currents Induced pollen tube growth	Wu et al., 2010
Antioxidant defense	*Zea may* L.	Leaf	H_2_O_2_ increased the concentration of cytosolic Ca^2+^ in the protoplasts of mesophyll cells and the expression of the calmodulin 1 (*CaM1*) gene and CaM content in leaves Enhanced the expression of the antioxidant genes	Hu et al., 2007

### Interaction in abiotic stress

Clearly, correlations also exist between H_2_O_2_ and Ca^2+^ in response to abiotic stresses in plants (Table 6). Shoresh et al. (2011) investigated that supplemental Ca^2+^ had a significant effect on H_2_O_2_ metabolism and regulating leaves and roots growth in maize under salt stress. The authors indicated that extracellular Ca^2+^ may modulate endogenous H_2_O_2_ levels through activating polyamine oxidase activity. Also, salt stress may induce H_2_O_2_ accumulation in Ca^2+^-dependent salt resistance pathway in *Arabidopsis thaliana* roots (Li et al., 2011). Moreover, Lu et al. (2013) suggested that exogenous H_2_O_2_ and Ca^2+^ may mediate root ion fluxes in mangrove species under NaCl stress. Obviously, H_2_O_2_ may interact with Ca^2+^ under salt stress in plants through mediating root ion balance, increasing antioxidant enzymatic activity and up-regulating the expression of related genes. Moreover, H_2_O_2_ and Ca^2+^ signaling were also involved in ABA responses to drought stress in *Arabidopsis thaliana* through Ca^2+^-dependent protein kinase8 (CPK8) which could regulate catalase3 (CAT3) activity mediating stomatal movement (Zou et al., 2015). In addition, Qiao et al. (2015) reported that a Ca^2+^-binding protein (rice annexin OsANN1) could enhance heat stress tolerance by modulating H_2_O_2_ production. Over production of H_2_O_2_ induced by heat stress increased *OsANN*1 expression and up-regulated the level of *SOD* and *CAT* expression, which constructed a signaling mechanism for stress defense in plants (Qiao et al., 2015). Until now, the signaling crosstalk between H_2_O_2_ and Ca^2+^ may regulate various responses to abiotic stresses in plants. It may be connected with the regulation of antioxidant system. Thus, the interaction between H_2_O_2_ and Ca^2+^ may increase antioxidant enzyme activities such as APX, SOD, and GR. These antioxidant enzymes may alleviate stress damages in plants. In addition, the crosstalk between H_2_O_2_ and Ca^2+^ could regulate gene expression level and induce protein interactions.

**Table 6:** **Reports on interaction between H_2_O_2_ and Ca^2+^ involved in abiotic stresses in plants**.

**Stress**	**Plant species**	**Tissue**	**Crosstalk between H_2_O_2_ and Ca^2+^ mediated effects**	**References**
Salt	*Bruguiera gymnorrhiza* L. *Kandelia candel* L.	Root Leaf	Mediated root ion flux Increased K^+^ flux and Na^+^/H^+^ antiport	Lu et al., 2013
	*Arabidopsis*	Root	Increased NADPH/NADP^+^,G6PDH activity Up-regulated expression of PM H^+^-ATPase gene	Li et al., 2011
Drought	*Zea may* L.	Root	Increased root viability Decreased membrane leakage Increased chlorophyll content Increased peroxidase activity	Shoresh et al., 2011
	*Arabidopsis*	Seedling	Induced stomatal closure Mediated protein interaction between CPK8 and CAT3	Zou et al., 2015
Heat	*Oryza sativa* subsp. japonica	Seedling	Up-regulated *OsANN1* expression Enhanced the level of *SOD, CAT* expression	Qiao et al., 2015

It appears that the interrelationship between H_2_O_2_ and Ca^2+^ may be involved in various aspects of plant growth and development processes and abiotic stress responses. In fact, the change of Ca^2+^ concentration is closely related to H_2_O_2_ burst in plant cells. The combination of H_2_O_2_ and Ca^2+^ may play crucial roles in plants. Different plants even different parts of the same plant may have different modulation mechanisms. Thus, relationship between H_2_O_2_ and Ca^2+^ signaling in plants is very complex. The interplay of H_2_O_2_, Ca^2+^ and its mechanism need to be illustrated clearly in the future.

## Crosstalk among H_2_O_2_, No and Ca^2+^

**Figure 3:**
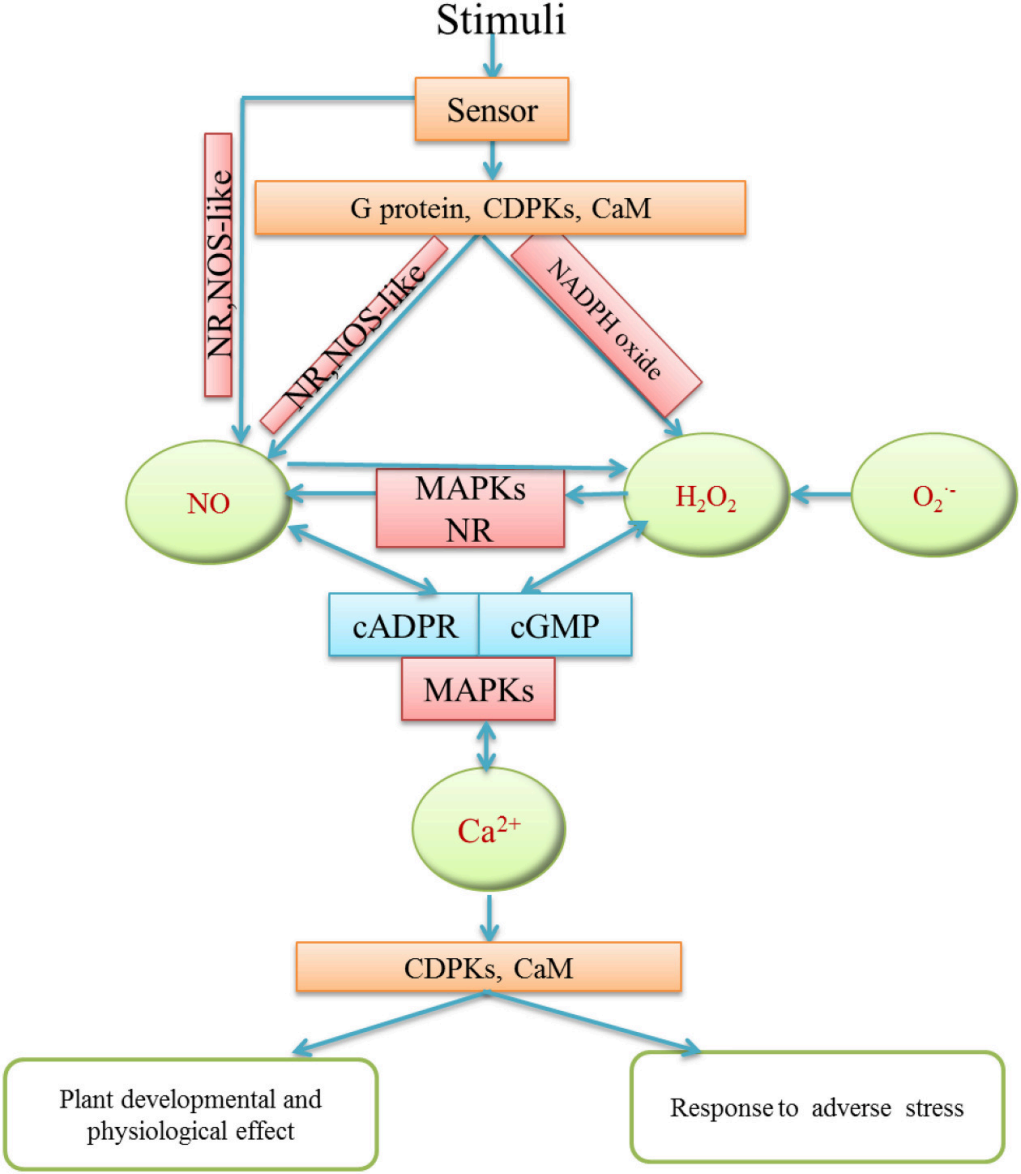
**Schematic model of the interaction among H_2_O_2_, NO, Ca^2+^ in different plant physiological and defense processes**. H_2_O_2_, NO and Ca^2+^ may receive various stimuli through signaling sensors. They might interact via cross-regulation and transduce signaling to downstream molecules by activating phosphokinase like MAPKs, or relative enzyme activity in order to regulate plant development and growth and abiotic stress responses.

It has been suggested that there is a connection among H_2_O_2_, NO, and Ca^2+^ in plants. H_2_O_2_, NO, and Ca^2+^ may act as essential signaling molecules which may form a complex signaling network to regulate different developmental and physiological processes in plants (Figure 3). For instance, during adventitious rooting of mung bean, Ca^2+^ signaling played a pivotal role and functioned as a downstream molecule of H_2_O_2_ and NO signal pathway (Li and Xue, 2010; Figure 3). Similarly, there is a possible relationship among H_2_O_2,_NO and Ca^2+^/CaM during adventitious rooting in marigold explants (Liao et al., 2012a). The authors found that exogenous NO and H_2_O_2_ promoted adventitious root development in marigold explants through increasing endogenous Ca^2+^ and CaM levels. Moreover, H_2_O_2_, NO and Ca^2+^ were also involved in oligochitosan-induced programmed cell death in tobacco suspension cells (Zhang et al., 2012). Pharmacological experiments revealed that Ca^2+^ signaling induced NO accumulation through inducing H_2_O_2_ generation during stomatal closure in *Arabidopsis* guard cells (Li et al., 2009). Furthermore, Wang et al. (2011) suggested a functional correlationship among H_2_O_2_, calcium-sensing receptor (CAS) and NO in Ca^2+^-dependent guard cell signaling. It was shown that CAS may transduce Ca^2+^ signaling through activating its downstream target NO and H_2_O_2_ signaling pathway (Wang et al., 2011). Therefore, it is thus clear that the interplay of H_2_O_2_, NO, and Ca^2+^ may have an significant effect on plant growth and physiological processes through promoting cell proliferation, controlling cell metabolism, meanwhile, regulating modes of cell death. Moreover, Vandelle et al. (2006) has reported that NO and H_2_O_2_ synthesis could also act upstream to increase cytosolic Ca^2+^ concentration during hypersensitive response (HR) through activating plasma membrane- and intracellular membrane-associated Ca^2+^ channels. Besides, the interaction among H_2_O_2_, NO, and Ca^2+^ signaling may regulate ABA-induced antioxidant defense in maize (Ma et al., 2012). Obviously, the mutual effect among H_2_O_2_, NO and Ca^2+^ may increase antioxidant system and induce disease defense in plants.

Furthermore, the interplay among H_2_O_2_, NO, and Ca^2+^ also have an effect on abiotic stress response in plants. For example, Lang et al. (2014) reported that NO likely interacted with Ca^2+^ and H_2_O_2_ in *Aegiceras corniculatum* to up-regulate Na^+^/H^+^ antiport system of plasma membrane under salt stress. There were species-specific interactions between H_2_O_2_, Ca^2+^, NO, and ATP in salt-induced reduction of K^+^ efflux (Lang et al., 2014). Moreover, there was a crosstalk among H_2_O_2_, NO, and Ca^2+^ when *Ulva compressa* exposed to copper excess and the interaction had a significant effect on transcriptional activation of target genes (Alberto et al., 2012). The H_2_O_2_-induced NO generation could be inhibited by Ca^2+^ channel blockers, implicating that Ca^2+^ may mediate the effect of H_2_O_2_ on NO production. Furthermore, Ca^2+^ release through different type of Ca^2+^ channels was also shown to be activated by NO and H_2_O_2_ (Alberto et al., 2012; Figure 3). The interrelationship between H_2_O_2_, NO and Ca^2+^ may provide additional layers of responses to abiotic stresses through controlling ion transport, increasing antioxidant enzyme activities and affecting expression of resistance genes, indicating a feedback mechanism between H_2_O_2_, NO and Ca^2+^ under abiotic stresses. In a word, the combination of these findings strongly supports the view that there has an interaction among H_2_O_2_, NO, and Ca^2+^ signaling pathway in plant growth, development and abiotic stress responses. During signaling transduction, Ca^2+^ signaling could be activated by H_2_O_2_ and NO; it could also regulate H_2_O_2_ and NO signaling. Ca^2+^ may act as a point of signaling convergence between H_2_O_2_ and NO signaling pathways in plants. However, the network of H_2_O_2_, NO, and Ca^2+^ seems to be intricate and multidimensional. Therefore, considerably more work will need to be done to determine the interaction among H_2_O_2_, NO and Ca^2+^ signaling in plants.

## Conclusion

H_2_O_2_ was once considered as a poisonous molecule in plants. Based on current studies, H_2_O_2_ may be a vital signaling molecule which controls plant growth and development. Interestingly, NO and Ca^2+^ which also act as the key component of signaling transduction in plants seem to be as upstream or downstream signaling molecules of H_2_O_2_. Meanwhile, H_2_O_2_ modulates NO and Ca^2+^ signaling pathways. There is a complex interactive network among H_2_O_2_, NO, and Ca^2+^ in plants. Moreover, the interplay among them has functional implications for regulating developmental and physiological processes which may increase the possibility of signal reception and transduction in plants. Future work will need to focus on the molecular mechanism of the interplay among H_2_O_2_, NO, and Ca^2+^ during signaling transduction in plants.

## Author contributions

LN wrote the paper. WL provided the idea and revised the paper.

### Conflict of interest statement

The authors declare that the research was conducted in the absence of any commercial or financial relationships that could be construed as a potential conflict of interest.
